# Anthocyanin pigment stability of *Cornus mas*–Macrocarpa under treatment with pH and some organic acids

**DOI:** 10.1002/fsn3.542

**Published:** 2017-11-20

**Authors:** Farideh Babaloo, Rashid Jamei

**Affiliations:** ^1^ Plant Physiology Faculty of Science Urmia University Urmia West Azerbaijan Iran; ^2^ Biology Department Faculty of Science Urmia University Urmia West Azerbaijan Iran

**Keywords:** Anthocyanin, blueberry, color, copigment, pH

## Abstract

The use of colors in food industry is essential for the creation of new products or their improvement. As an important pigment group, anthocyanin could be used as a natural coloring pigment in foods. This study aims at exploring strategies that result in color stability of anthocyanin in pear‐shaped variety of blueberry (*Cornus mas*–Macrocarpa). In this study, the effects of different pH values (1, 2, 3, 4) as well as various concentrations (0, 120, 240, 480, 960 mg/L) of five copigments, including tannic, caffeic, benzoic, and coumaric acids, on anthocyanin copigment complexes (ratio 1:1) were investigated. The studied copigments were tannic, caffeic, benzoic, and coumaric acids. Anthocyanin was influenced by the highest concentration of 960 mg/L copigment in the presence of different pHs. Five groups were considered, one of which contained anthocyanin without copigment and the rest consisted of copigments. To evaluate the response of copigmentation through spectrophotometer, absorbance from samples was measured after 30 min of adding copigment to anthocyanin in the range of 450–600 nm wavelengths. The results showed that caffeic acid possessed the greatest anthocyanin stability compared to other copigments and it was the best copigment. An increase in the concentrations of copigments led to a higher level of anthocyanin stability and changes in hyperchromic and bathochromic. Moreover, the results revealed that the strongest hyperchromic effect for all organic acids was observed in pH 2, and the strongest bathochromic changes were observed in pH 4.

## 1 INTRODUCTION

Pear‐shaped variety of blueberry (*Cornus mas*–Macrocarpa) belongs to the Cornaceae family; a shrub to a height of about 5–8 m with tiny leaves that grows in the vast region of Europe and Asia especially Arasbaran (Kaleibar) forests(Hassanpour, Hamidoghli, & Samizadeh, 2012, 2013). Blueberry contains tannins and organic acids, anthocyanins, carotenoids, and antioxidant melatonin. Besides, this fruit's extract is antidiabetic, antibacterial, anticancer, anti‐ pain and fever, and antioxidant while it has counter‐oxidative properties due to its ascorbic acid (Mirbadalzadeh & Shirdel, 2012; Tsuda, 2012).

Anthocyanins are a large subgroup of flavonoids responsible for red, purple, and blue colors in flowers, fruits, and vegetables (Chandrasekhar, Madhusudhan, & Raghavarao, 2012; Malaj, de Simone, Quartarolo, & Russo, 2013). They play an important role in the treatment of high blood pressure, liver disorders, diarrhea, and urinary problems such as kidney stones, urinary tract infections, and the common cold (Ercisli et al., 2011; Li et al., 2016; West, Ma, Deng, Jensen, & Su, 2012). Anthocyanins as a natural food coloring are an excellent option for food industry, but their very low stability in food matrix has caused some limitations (Jing et al., 2012; Pineda‐Vadillo et al., 2017) .Increasing the concentration of anthocyanins leads to greater stability and increased color intensity (several times) (Su et al., 2016).

Solvents, storage temperature, pH, concentration, structure of anthocyanins, copigments, oxygen, light, the use of polyphenols, presence of enzymes, and other materials connected to them significantly affect the stability of anthocyanin (Chung, Rojanasasithara, Mutilangi, & McClements, 2016; Jiao et al., 2016). Anthocyanins are more stable in acidic environments with lower pH compared to alkaline solvents with higher pH and are mainly found in the form of red falvylum. Adding copigment results in a significant color maintenance in anthocyanins which is due to copigment role in preventing the formation of a colorless falvylum cation (Andersen & Markham, 2005). Gauche, Malagoli, and Bordignon Luiz (2010) studied the effects of pH on the stability of anthocyanins in combination with organic acids. In this study, anthocyanins extracted from grapes (*Vitis vinifera* L.) were combined with four organic acids (tannic, caffeic, ferulic and gallic acid) and their stability was measured at different pHs (1, 2, 3, 3.3, 3.5, 3.7, 4, and 4.5). They found that most stability in all acids were at pH 3.3.

Copigmentation is an available natural tool to enhance anthocyanin color food of products; a color that can be stabilized and increased by intensity after adding different plant extracts rich of copigments (Ko, Lee, Sop Nam, & Gyu Lee, 2016; Zozio, Pallet, & Dornier, 2011). Similar to other anthocyanins reactions, copigmentation reactions are also affected by pH, temperature, concentration, and molecular structure (Sari, 2015; Wang, Shen, & Chen, 2013; Weber, Boch, & Schieber, 2017). Copigments are individually colorless, but they get an increased color intensity and solvent stability when added to the anthocyanin solvent (Heras‐Roger, Alonso‐Alonso, Gallo‐Montesdeoca, Díaz‐Romero, & Darias‐Martín, 2016). Copigments are an electron‐rich system able to communicate with falvylum ion that is electron poor (Weber et al., 2017). The association protects the water falvylum ions from nucleophilic attack (Gris, Ferreira, Falcão, & Bordignon Luiz, 2007; Pina, 2014). Most studied group from copigments is flavonoids. Besides, phenolic acids such as hydrosynamic acids and hydroxy benzoic acids are thoroughly investigated in copigments phenomenon and has great effect on the stability of anthocyanins (Castaneda‐Ovando, De Lourdes Pacheco‐Hernández, Páez‐Hernández, Rodríguez, & Galán‐Vidal, 2009; Willstatter & Zollinger, 2008). Zhang, Ma, Zhao, and Mu (2009) studied the effect of citric acid copigmentation on potato peel anthocyanins through thermodynamic measurements and found that citric acid enhances anthocyanin stability.

With reference to the findings from similar studies in the literature, it was found out that the colors were fortified and became stronger in the copigmentation of phenolic acids with anthocyanins.(Pineda‐Vadillo et al., 2017; Sun, Cao, Liao, & Hu, 2010; Tierno & Ruiz De Galarreta, 2016).

Color is a crucial criterion of a food product for clients. When manufacturing situations had been taken into consideration, in the course of storage, color bleaching and/or losses in ingredients could occur. For that reason, food‐coloring additives are used to augment coloration losses or enhance coloration. The most vital issue of concern among natural colorants is anthocyanin. However, distinctly low stability of anthocyanins in comparison to artificial dyes has restricted their use as natural colorants. Copigments are a group of colorless or pale substances which result in reinforcement of anthocyanins by making complex and thereby increasing the tone of anthocyanins color. Even though they are typically colorless, they may be delivered to solutions containing anthocyanins. Their color turns out to be more potent and the balance of the color increases their trade potentials. For these reasons, the purpose of this study was to stabilize the color of anthocyanin pigments through copigmentation methods. In this research, the effect of various pH values and different concentrations of copigments on the stability of anthocyanins in pear‐shaped variety of blueberry (*C. mas*–Macrocarpa) were investigated.

## 2 MATERIALS AND METHODS

### 2.1 Sample preparation and anthocyanin extraction

Agriculture Research Center of Kaleybar in East Azerbayjan province in Iran supplied the main sample of *C. mas*–Macrocarpa in September 2015. Department of Botany and Herbal Medicine at Urmia University, Urmia, Iran was in charge of pharmacognostic verification of the plant. Finally, the voucher specimens were deposited at the institute herbarium and received the code no: 4560.

The method consisted of extracting the anthocyanin using 96% ethanol and 0.1% HCl (Wrolstad et al., 2005). Then, 100 g of the specimen was mixed with 100 ml of extraction solvent to an extent that it was completely crushed and homogenous. Next, it was stirred with a magnetic stirrer for 4 hr. Later, the solvent was filtered by Buchner funnel and Whatman No.1 filter and diluted it to 500 ml. Afterwards, the resultant solvent was centrifuged at 8,000 g for 30 min and then, upper colored and clear solvent was gathered for the experimentations. At the end, the samples were kept in sealed vials for a period of 1 day at 4°C temperature (Skrede, Wrolstad, & Durst, 2000).

### 2.2 The preparation of anthocyanin‐copigment complex and pH treatment

Four types of organic acids were used comprising benzoic, tannic, caffeic, and coumaric acids for this study. The effect of the type of copigment on anthocyanin stability was evaluated by the same concentration (960 mg/L) and copigment‐anthocyanin ratio (1:1) for all copigments. Five groups were formed consisting of the control group (Ι) that got no copigment and treatment groups (ΙΙ–IV) whom we administered various kinds of copigments. Solvents’ absorbance was measured half an hour after the addition. Organic acids tannic, coumaric, caffeic, and benzoic acids were added and studied to the anthocyanins at a ratio of 1:1. The optical absorption rate was measured to assess copigmentation reactions spectrophotometerically at wavelengths of 450 and 650 nm (Biowave, S2100, UK) (Gauche et al., 2010; Giusti & Wrolstad, 2003). Five varying concentrations of each copigment (0, 120, 240, 480, and 960 mg/L) with the ratio of 1:1 to anthocyanin were used (Gauche et al., 2010). Next, the mixture of copigments and anthocyanin was prepared and after 30 min, the absorption level was measured at pH 3.5. Finally, the effects of various pH values, namely 1, 2, 3, and 4, were evaluated on the copigmentation reaction with the dissimilar concentrations of each copigment as well (Lambert, Asenstorfer, Williamson, Iland, & Jones, 2011; Wang & Xu, 2007).

### 2.3 Statistical analysis

All experimentations were performed in triplicate. The results were obtained in the form of mean and standard deviation (*SD*) of the means. One‐way ANOVA analysis and Tukey's test with the probability of *p ≤ *.05 was utilized to assess the difference between the treatments. SPSS 21 and Microsoft Excel were used to plot the diagrams and analyze the statistics.

## 3 RESULTS AND DISCUSSION

**Figure 1:**
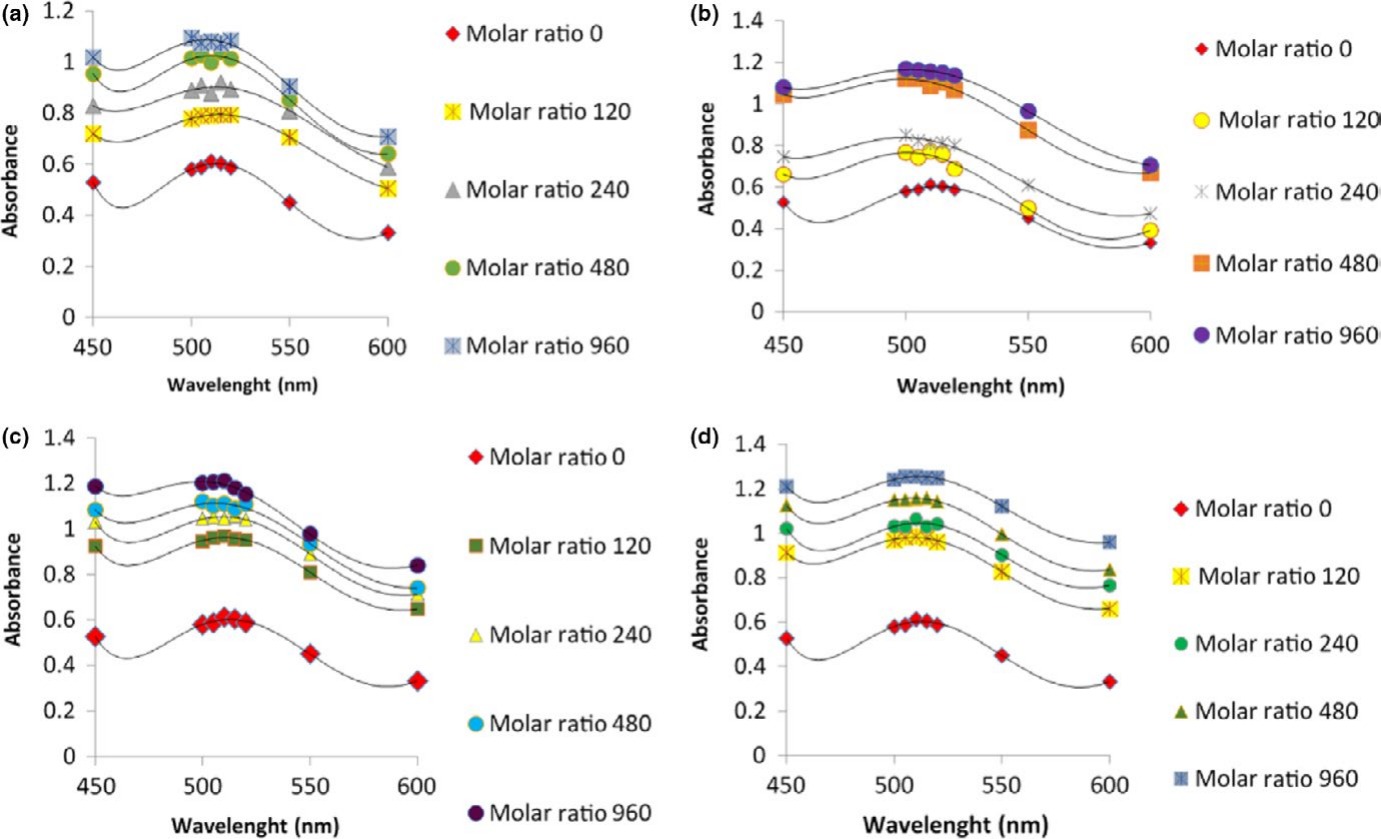
Absorption spectra of various anthocyanins with different concentrations at pH 3.5 for pear‐shaped variety of blueberry. (a) Tannic acid, (b) benzoic acid, (c) coumaric acid, (d) caffeic acid. The data are the mean value for three replications ± standard deviation

Among the factors affecting the copigmentation reactions is copigmental intensity that is added to anthocyanin. Copigmentation intensity depends on the molar ratio of anthocyanin to copigment (Jamei & Babaloo, 2017; Sari, 2015). The study revealed that the concentration of anthocyanin and copigment both affect copigmentation. Increasing the concentration of anthocyanins enhances bathchromic absorption. Increase in absorption depends on the molar ratio of anthocyanins to copigment (Jamei & Babaloo, 2017; Sari, 2015). Anthocyanin concentration was considered stable in the present research and by changing the concentration of copigment, the results were evaluated based on changes at the concentration of copigment. Due to the fact that anthocyanins were stable, it could be deduced that the ratio of bathochromic shift and hyperchromic effect depends on the concentration of copigment. Copigment used in this study consisted of coumaric, tannic, caffeic, and benzoic acids. In the current research, effects of five different concentrations of the aforementioned copigments (0, 120, 240, 480, and 960 mg/L) were assessed at stable pH value of 3.5 (Gauche et al., 2010; Jamei & Babaloo, 2017). and the ratio of 1:1 was used for each concentration of anthocyanins. The results were as the following graphs (Figure 1).

**Figure 2:**
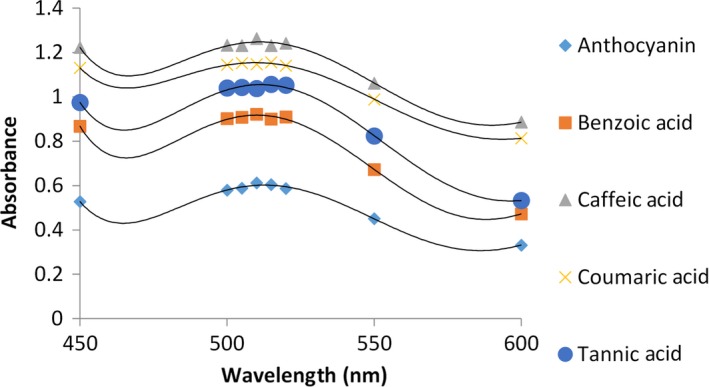
Comparison of absorption spectra of anthocyanin with different copigments (960 mg/L) and without copigment at pH 3.5 for pear‐shaped variety of blueberry. The data are the mean value for three replications ± standard deviation

The results showed that the most stability and impact of concentration is observed in 960 mg/L and increasing concentrations of copigments, enhances absorption and stability of anthocyanins along with hyperchromic and bathochromic changes. Anthocyanin‐copigment complex showed a higher level of stability compared to anthocyanin individually and adding materials as copigments results in durability and stability of anthocyanins in bad conditions and it also is an inhibiting factor of anthocyanin pigments’ destruction. In pear‐shaped variety of blueberry (*C. mas*–Macrocarpa), caffeic acid at a concentration of 960 mg/L caused greater stability in anthocyanins compared to other copigments and benzoic acid at a concentration of 960 mg/L has less impact on stability (Figure 2).

**Figure 3:**
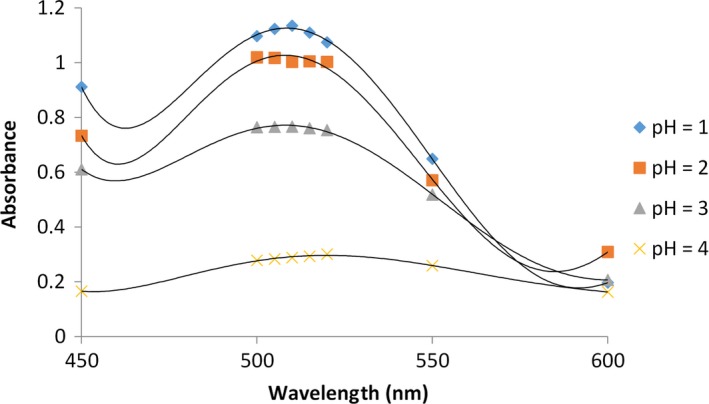
Diagram of the effects of different pH values on anthocyanin in blueberry; the data are the mean value for three replications ± standard deviation

Another factor affecting copigmentation is pH. This article aimed at investigating the effect of 1, 2, 3, and 4 pHs by adding tannic, caffeic, benzoic and coumaric acid at the ratio of 1:1. Anthocyanins are more stable in acidic environments with lower pH compared to alkaline solvents. The findings of this study revealed that pH increase causes a high level of destruction of anthocyanins in the samples. The present research also explored the effect of pH on anthocyanin‐copigment complex. The findings indicated that the highest anthocyanins absorption rate and also the highest stability of anthocyanin‐copigment complex occur in the lowest pH value (Figure 3). Table 1 reports the level of bathochromic and hyperchromic after the addition of organic acids to anthocyanin solvent in maximum absorption of anthocyanins in four differing pH of blueberry (*C. mas*–Macrocarpa).

**Table 1:** The level of bathochromic and hyperchromic in maximum absorption of anthocyanins of pear‐shaped variety of blueberry (*Cornus mas*–Macrocarpa) of tannic, coumaric, caffeic, and benzoic acids in four differing pH

pH	Anthocyanin		Tannic acid		Coumaric acid		Caffeic acid		Benzoic acid	
	∆*A*	∆λ (nm)	∆*A*	∆λ (nm)	∆*A*	∆λ (nm)	∆*A*	∆λ (nm)	∆*A*	∆λ (nm)
1	135/1	510	482/0	0	484/0	5	675/0	5	655/0	5
2	118/1	505	562/0	5	49/0	0	721/0	0	432/0	5
3	667/0	51	27/0	5	207/0	0	035/0	0	015/0	5
4	201/0	520	09/0	10	067/0	10	**001/0**	5	088/0	10

∆A=0.721

∆A=0.001.

According to the obtained values in pear‐shaped variety of blueberry (*C. mas*–Macrocarpa), the highest level of hyperchromic of all organic acids was observed in pH 2 (∆*A* = 0.721), but these variations were not associated with changes bathochromic and most changes in bathochromic was observed of pH 4. The least level of effect of hyperchromic was observed in anthocyanin‐caffeic acid complex (∆*A* = 0.001). Bathochromic changes were not found in pHs 3 and 2, however, most changes were observed in bathochromic of pH 4. In pear shaped variety of pHs 1 and 2, the most stable copigment belonged to anthocyanin‐caffeic acid complex. In pH 4, the most stable copigment was anthocyanin‐tannic acid complex in which the destruction of anthocyanin increased and the color of anthocyanin‐copigment complex was severely weakened. The results revealed that highest stability of anthocyanin–copigment complex was observed in pH 1 and by increasing pH from 1 to 4, absorption is decreased due to the decrease in cation of falvylum crude extract and more anthocyanins are destructed. In highly acidic solvents (pHs 3 and 4), lots of hemiketals appear colorless and chalcone forms could change into flavylum cations that are quinoid through the formation of copigmentation complexes and as a result, produce significant color, besides, in these solvents, all anthocyanins are in flavylum colored form (Pina, 2014; Weber et al., 2017).

Gras, Bogner, Carle, and Schweiggert (2016) analyzed the coloration depth and stability of black carrot anthocyanins by way of intermolecular copigmentation with the addition of chlorogenic acid, and an aqueous phenolic‐rich green coffee bean extract at numerous anthocyanin copigment ratios on heating. They found out that the hyperchromic copigmentation effect fixated the concentration of delivered co‐pigments, which was followed by an absorption increase in over one fifth. After adding anthocyanin copigments, heat stability substantially improved (Gras et al., 2016).

Recently, Jamei & Babaloo (2017) investigated anthocyanin stability of *C. mas*–Yulyush by means of pHs and copigment treatments. They proved that in higher pH value degradation of anthocyanin and in comparison to other copigments, caffeic acid had a profound preventive impact and benzoic acid had the lowest hyperchromic and bathochromic changes.

(Malaj et al., 2013) revealed that anthocyanin–copigment leads to an increase in bath‐ and hyperchromic effect (Malaj et al., 2013).

## 4 CONCLUSION

The findings of the current article on pear‐shaped variety of blueberry (*C. mas*–Macrocarpa) showed that most hyperchromic effect all organic acids was seen of pH 2. These changes were not associated with changes of bathochromic and the most changes were observed in pH 4. The least effect of hyperchromic was recorded in anthocyanin‐caffeic acid complex. Bathchromic changes were not significant in pHs 2 and 3, but it was the highest in pH 4. In pear‐shaped variety of pHs 1 and 2, the most stable copigment was anthocyanin‐caffeic acid complex. In pH 4, the most stable copigment was anthocyanin‐tannic acid complex in which the level of anthocyanins destruction increased and anthocyanin‐copigment complex was severely weakened in color. The results of this research may be beneficial for improving anthocyanin stability from the point of color and could be utilized in food and pharmaceutical industries considering the necessity to use nonsynthetic colors and the significance of natural colors in meals productions.

## CONFLICT OF INTEREST

None declared.
